# MACE for Diagnosis of Dementia and MCI: Examining Cut-Offs and Predictive Values

**DOI:** 10.3390/diagnostics9020051

**Published:** 2019-05-06

**Authors:** Andrew J. Larner

**Affiliations:** Cognitive Function Clinic, Walton Centre for Neurology and Neurosurgery, Liverpool L9 7LJ, UK; a.larner@thewaltoncentre.nhs.uk or A.J.Larner@thewaltoncentre.nhs.uk

**Keywords:** diagnosis, dementia, mild cognitive impairment, Mini-Addenbrooke’s Cognitive Examination

## Abstract

The definition of test cut-offs is a critical determinant of many paired and unitary measures of diagnostic or screening test accuracy, such as sensitivity and specificity, positive and negative predictive values, and correct classification accuracy. Revision of test cut-offs from those defined in index studies is frowned upon as a potential source of bias, seemingly accepting any biases present in the index study, for example related to sample bias. Data from a large pragmatic test accuracy study examining the Mini-Addenbrooke’s Cognitive Examination (MACE) were interrogated to determine optimal test cut-offs for the diagnosis of dementia and mild cognitive impairment (MCI) using either the maximal Youden index or the maximal correct classification accuracy. Receiver operating characteristic (ROC) and precision recall (PR) curves for dementia and MCI were also plotted, and MACE predictive values across a range of disease prevalences were calculated. Optimal cut-offs were found to be a point lower than those defined in the index study. MACE had good metrics for the area under the ROC curve and for the effect size (Cohen’s d) for both dementia and MCI diagnosis, but PR curves suggested the superiority for MCI diagnosis. MACE had high negative predictive value at all prevalences, suggesting that a MACE test score above either cut-off excludes dementia and MCI in any setting.

## 1. Introduction

The Mini-Addenbrooke’s Cognitive Examination (MACE) is a shortened version of the Addenbrooke’s Cognitive Examination-Revised (ACE-R) and ACE-III developed by Mokken scaling analysis of these longer instruments [1]. MACE comprises tests of attention, memory (7-item name and address), verbal fluency, clock drawing and memory recall (score range 0–30, impaired to normal), and takes between 5–10 min to administer.

In the index MACE study, two cut-off points were identified in the cohort examined (*n* = 242; Alzheimer’s disease 28, behavioural variant frontotemporal dementia 23, primary progressive aphasia 82, corticobasal syndrome 21, controls 78): ≤25/30 had high sensitivity (0.85) and high specificity (0.87); and ≤21/30 had high specificity (1.00), and hence an abnormal score was almost certain to have come from a dementia patient. MACE was found to be more sensitive than the Mini-Mental State Examination (MMSE) and less likely to have ceiling effects [1].

The general applicability of these MACE cut-offs for the diagnosis of dementia and mild cognitive impairment (MCI) has not been widely examined. A Spanish translation administered to a cohort of mixed dementia patients and controls (*n* = 175) with relatively low educational experience found that a cut-off between 16/30 and 17/30 had optimal sensitivity (0.867) and specificity (0.870) for dementia diagnosis [2].

MACE was adopted in the author’s practice, based in a dedicated Cognitive Function Clinic located at a regional neuroscience centre in the northwest United Kingdom in June 2014 [3], and has been routinely used since then. Access to a large dataset has provided the opportunity to examine a variety of parameters not hitherto examined. The aims of this study were:

- To determine optimal MACE cut-off points for the diagnosis of dementia and MCI, with the anticipation that cut-offs optimising sensitivity would also minimise false negative rate (FNR) and optimise negative predictive value (NPV) whilst cut-offs optimising specificity would minimise false positive rate (FPR) and optimise positive predictive value (PPV);
- To plot receiver operating characteristic (ROC) and precision recall (PR) curves for dementia and MCI, and to calculate areas under the ROC curves and Q* index (a measure of diagnostic value);
- To calculate MACE effect sizes (Cohen’s d) for diagnosis of dementia and MCI;
- To calculate MACE predictive values across a range of disease prevalences.

## 2. Methods

Consecutive new patient referrals administered the MACE were included, seen over the period June 2014–December 2018 (inclusive), including data reported in previous studies [3,4,5]. Other than those with a pre-existing diagnosis of dementia, there were no exclusion criteria. As previously detailed [3,4,5], criterion diagnosis of dementia or mild cognitive impairment was by judgement of an experienced clinician using standard diagnostic criteria (DSM-IV; Petersen); in those without evidence of cognitive impairment, a diagnosis of subjective memory complaint (SMC) was made. MACE scores were not used to make criterion diagnoses to avoid review bias. Subjects gave informed consent, and the study protocol was approved by the institute’s committee on human research.

MACE scores were plotted against diagnosis, and the Pearson 2 skewness coefficient (Sk2) was used to assess skew, where:

Sk2 = 3(mean – median)/standard deviation,

with values lying between –1 and +1 deemed acceptable for the elimination of floor or ceiling effects [6].

Various measures were calculated for all MACE cut-offs within the range deemed clinically sensible. Paired measures were: sensitivity (Sens, or recall) and specificity (Spec); positive and negative predictive values (PPV or precision, and NPV); positive and negative likelihood ratios (LR+ and LR–); and positive and negative clinical utility indexes (CUI+ and CUI–). Unitary or global measures were: correct classification accuracy (Acc); Youden index (Y, where Y = Sens + Spec – 1) [7]; predictive summary index (PSI, where PSI = PPV = NPV – 1) [8]; and identification index (II, where II = 2(Acc – 1) [9]. 

Two methods to optimise test cut-offs were examined [10,11], namely, maximising either Acc or Youden index.

In addition, various “number needed to” metrics were calculated: number needed to diagnose (NND, where NND = 1/Y); number needed to predict (NNP, where NNP = 1/PSI) [8]; and number needed to misdiagnose (NNM, where NNM = 1/(1 – Acc)) [12]. Also calculated were the “likelihood to be diagnosed or misdiagnosed” ratio (LDM, where LDM = NNM/NND or NNM/NNP) [13,14], and the “summary utility index” (SUI, where SUI = (CUI+ + CUI–)) [15].The multiplicative inverse of the latter—the “number needed for screening utility” (NNSU = 1/SUI) [15]—was compared to the “number needed to screen” (NNS) metric, the multiplicative inverse of the “identification index” (II) [9], both rounded to the next highest integer value since they represent numbers of patients.

ROC curves were plotted (false positive rate versus sensitivity) and areas under the curve (AUC ROC) were calculated and categorised according to the scale of Metz [16]. The Q* index, a measure of diagnostic value [17], was determined as the point in ROC space where the anti-diagonal intersected the ROC curve (i.e., where Sens = Spec). Precision recall (PR) curves [18] were also plotted, as these have been recommended over ROC curves when analysing highly skewed datasets [19]. The F measure, or F1 score—the harmonic mean of precision and sensitivity—was also calculated as a global measure of accuracy [20].

Effect sizes (Cohen’s d) were calculated as the difference of the means of diagnostic groups divided by the weighted pooled standard deviations of the groups [21]. Cohen’s d values were categorised according to Sawilowsky’s extension of Cohen’s rules of thumb [22].

Predictive values across a range of disease prevalence (Prev) were calculated from observed sensitivity and specificity at the maximum Youden index for dementia and MCI, specifically at prevalence rates of 5%, 10%, 20% and 40% using the standard formulae:

PPV = Sens × Prev/(Sens × Prev) + [(1 – Spec) × (1 – Prev)],

NPV = Spec × (1 – Prev)/[Spec × (1 – Prev)] + [(1 – Sens) × Prev].

## 3. Results

**Figure 1:**
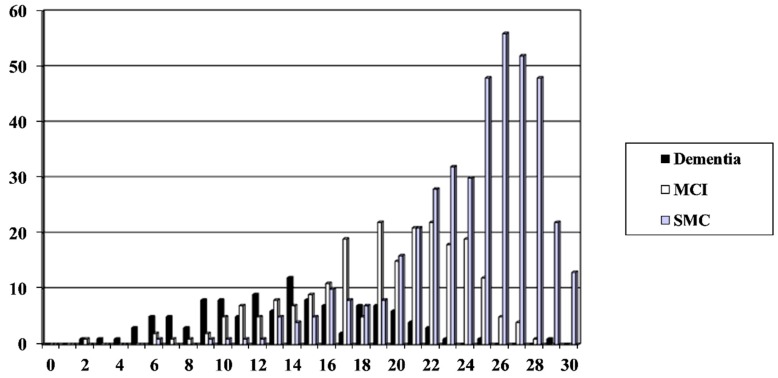
Mini-Addenbrooke’s Cognitive Examination (MACE) scores versus patient diagnosis (*n* = 755). MCI: mild cognitive impairment; SMC: subjective memory complaint.

A total of 755 patients were assessed with MACE (F:M = 352:403, 47% female; median age 60 years), of whom 114 were diagnosed with dementia (prevalence = 0.15) and 222 with MCI (prevalence = 0.29). The distribution of MACE scores by diagnosis, as seen in Figure 1, showed the anticipated unimodal negative skew (to the right, i.e., higher test scores = better performance). The Pearson 2 skewness coefficient (Sk2) was −0.48, suggesting that the population sampled was not from a normal distribution, although, as the value was between −1 and +1, the presence of floor or ceiling effects was probably excluded.

For the diagnosis of dementia (Table 1 and Table 2), looking at all MACE cut-off values, the optimal cut-off determined by maximal Youden index was ≤20/30 (sensitivity 0.91, specificity 0.71). By maximal correct classification accuracy the optimal cut-off was ≤14/30 (sensitivity 0.59, specificity 0.92). This latter cut-off also had the maximal values of PSI and LDM (Table 3), the latter also found at ≤15/30, which was also the cut-off for the maximal value of SUI and F measure.

For the diagnosis of MCI (Table 4 and Table 5), looking at all MACE cut-off values, the optimal cut-off determined by maximal Youden index was ≤24/30 (sensitivity 0.90, specificity 0.57). By maximal correct classification accuracy the optimal cut-off was ≤19/30 (sensitivity 0.47, specificity 0.88). Both cut-offs coincided with the maximal values of LDM (Table 6), whereas maximum PSI and F measure were at the same cut-off as maximal Youden index (cf. diagnosis of dementia), whilst maximal SUI was at ≤22/30 and ≤21/30.

Comparison of the values for NNS and NNSU (Table 3 and Table 6) showed negative values of NNS (which are clinically meaningless) when identification index (II) had negative values, but this was not the case with NNSU, since by definition SUI cannot be <0. 

**Figure 2:**
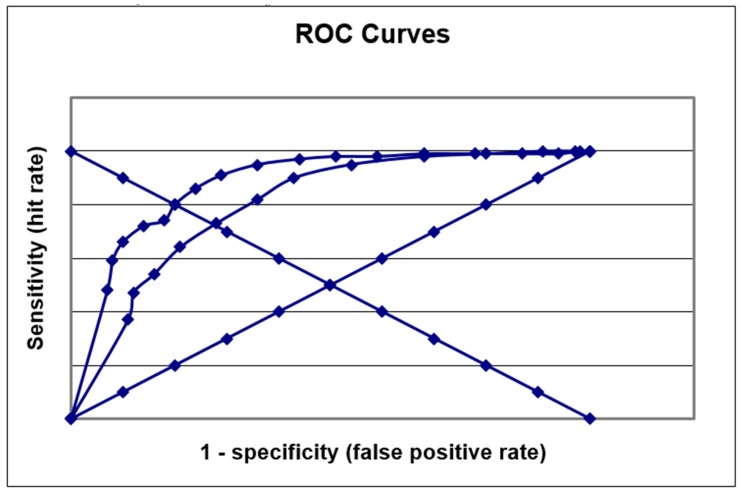
MACE receiver operating characteristic (ROC) plots for the diagnosis of dementia (upper; area under the curve (AUC) = 0.89) and MCI (lower; AUC = 0.81) with chance diagonal (*y* = *x*) and anti-diagonal (*y* = 1 − *x*) lines.

**Figure 3:**
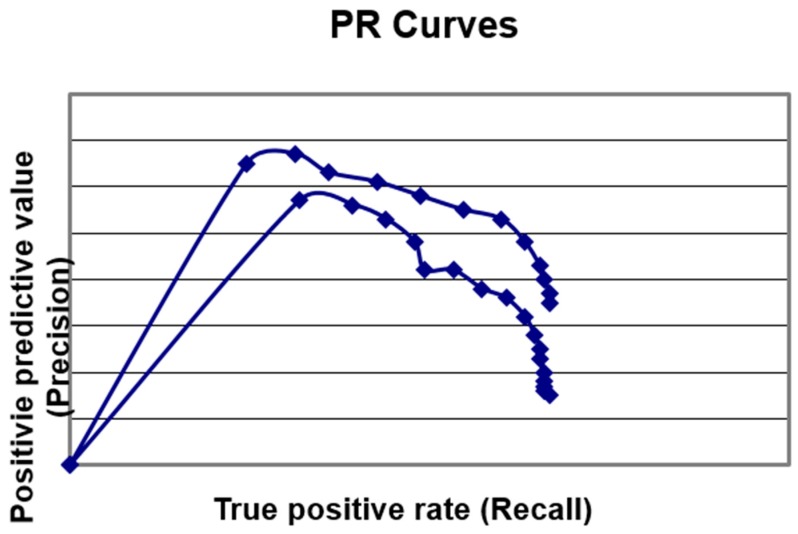
MACE precision recall (PR) curves for the diagnosis of dementia (lower) and MCI (upper); note the reversal of position versus ROC curves.

A ROC curve was plotted for the diagnosis of dementia and MCI (Figure 2). The AUC ROC was 0.89 (95% confidence interval (CI) 0.86–0.92) for the diagnosis of dementia and 0.81 (95% CI 0.77–0.84) for the diagnosis of MCI, hence the AUC ROC values were categorised as good [16] for the diagnosis of both dementia vs. no dementia and MCI vs. SMC. The Q* index for dementia was 0.8 (comparable to the value of 0.76 found in a previous study examining the first 135 patients in this cohort [3]) and for MCI it was 0.73. PR curves (Figure 3) did not show the desired approximation to the top-right corner of the graph, reflecting the relatively poor PPV of MACE, but did show greater area under the curve for MCI versus dementia.

Effect sizes (Cohen’s d) were 1.74 for dementia and 1.13 for MCI, hence very large and large, respectively [22].

PPV and NPV for MACE calculated at prevalence rates of 5%, 10%, 20% and 40% using the sensitivity and specificity figures at the maximum Youden index showed high NPV (≥0.9) at all prevalences examined, but with less-impressive figures for PPV, optimal at higher disease prevalences (Table 7).

## 4. Discussion

This study of a large cohort of patients examined with the Mini-Addenbrooke’s Cognitive Examination suggested that the optimal test cut-offs differ slightly from those suggested in the index study, being a point lower for both high-sensitivity (≤24/30 vs. ≤25/30) and high-specificity (≤20/30 vs. ≤21/30) cut-offs. Even allowing for the objections raised to changing test cut-offs because of risk of bias [23], these findings suggest a possible need for cut-off revision when using MACE in general cognitive clinics, as well as for patient educational level [2].

ROC curves suggested that MACE had adequate accuracy for the diagnosis of dementia and MCI—a finding corroborated by the measure of effect size (Cohen’s d). The Q* index for dementia (0.80) was comparable to that found for other cognitive screening instruments [24], but the Q* index for MCI was lower (0.73). However, PR curves suggested better MACE performance (i.e., distinguishable classification performance) for the diagnosis of MCI than for dementia, consistent with findings in previous comparative studies of MACE with the Montreal Cognitive Assessment (MoCA)—a test designed specifically for MCI diagnosis (equivalent MACE performance) [25,26], and Free-Cog (superior MACE performance) [5]. The use of PR curves [18] in diagnostic test accuracy studies is worth emphasizing (to the author’s knowledge this is the first such use for dementia test accuracy studies), since these avoid some of the “optimism” of ROC curves (resulting from their combining test accuracy over a range of thresholds which may be both clinically relevant and clinically nonsensical) [27]. PR curves are more informative than ROC curves for skewed datasets [19]. Area under the PR curve may be calculated, although this is not straightforward [28], and visual interpretation may be adequate to denote better classification performance (as for ROC curves).

The comparison of NNS [9] and NNSU [15] in this study was also instructive, demonstrating some of the difficulties in working with reciprocals (multiplicative inverses). By definition, the identification index (II) ranges from −1 to +1, and hence NNS ranges from −∞ to +∞ [9]. As II approaches 0, values of NNS are inflated (e.g., Table 3, cut-off ≤24/30; Table 6, cut-off ≤27/30), and when II is negative then NNS also has a negative value. The latter finding is problematic from the clinical standpoint: “number needed to” metrics were originally designed to appeal intuitively at the individual level, so negative values (representing non-individuals?) are meaningless. The construction of SUI is such that by definition its range is from 0 to 2, and hence NNSU ranges from ∞ (no screening value) to 0.5 (perfect screening utility) [15], avoiding the problems encountered with II and NNS.

The high NPV (≥0.9) at all disease prevalences examined (0.05 to 0.4) suggests that a MACE test score above either cut-off excludes dementia and MCI in any setting. PPV was less impressive, but improved with increasing disease prevalence, suggesting a case-finding role for MACE in dedicated cognitive and memory clinics.

In summary, in addition to being quick, easy to use and score and acceptable to patients [1,3,4], when using appropriate cut-offs MACE is a sensitive test for the identification of cognitive impairment and for excluding dementia and MCI with scores below the cut-offs.

## Figures and Tables

**Table 1:** Diagnosis of dementia: paired measures of discrimination at various MACE cut-offs.

Cut-off	Sensitivity (= Recall); Specificity	False Positive Rate (FPR); False Negative Rate (FNR)	PPV (= Precision); NPV	LR+; LR−	CUI+; CUI−
≤29/30	1.00; 0.02	0.98; 0	0.15; 1.00	1.02; 0	0.15; 0.02
≤28/30	0.99; 0.06	0.94; 0.01	0.16; 0.97	1.05; 0.16	0.16; 0.05
≤27/30	0.99; 0.13	0.87; 0.01	0.17; 0.99	1.14; 0.07	0.17; 0.13
≤26/30	0.99; 0.22	0.78; 0.01	0.18; 0.99	1.27; 0.04	0.18; 0.22
≤25/30 *	0.99; 0.32	0.68; 0.01	0.20; 0.99	1.45; 0.03	0.20; 0.32
≤24/30	0.98; 0.41	0.59; 0.02	0.23; 0.99	1.66; 0.04	0.23; 0.41
≤23/30	0.98; 0.49	0.51; 0.02	0.25; 0.99	1.91; 0.04	0.25; 0.49
≤22/30	0.97; 0.56	0.44; 0.03	0.28; 0.99	2.23; 0.05	0.27; 0.55
≤21/30 *	0.95; 0.64	0.36; 0.05	0.32; 0.99	2.64; 0.08	0.30; 0.63
≤20/30	0.91; 0.71	0.29; 0.09	0.36; 0.98	3.11; 0.12	0.33; 0.70
≤19/30	0.86; 0.76	0.24; 0.14	0.38; 0.97	3.51; 0.19	0.33; 0.74
≤18/30	0.80; 0.80	0.20; 0.20	0.42; 0.96	4.03; 0.25	0.34; 0.77
≤17/30	0.74; 0.82	0.18; 0.26	0.42; 0.95	4.11; 0.32	0.31; 0.78
≤16/30	0.72; 0.86	0.14; 0.28	0.48; 0.95	5.24; 0.33	0.35; 0.82
≤15/30	0.66; 0.90	0.10; 0.34	0.53; 0.94	6.29; 0.38	0.35; 0.85
≤14/30	0.59; 0.92	0.08; 0.41	0.56; 0.93	7.11; 0.45	0.33; 0.86
≤13/30	0.48; 0.93	0.07; 0.52	0.57; 0.91	7.36; 0.55	0.27; 0.85

CUI+ and CUI−: positive and negative clinical utility indices; LR+ and LR−: positive and negative likelihood ratios; NPV: negative predictive value; PPV: positive predictive value. * Test cut-off established in index study (Hsieh et al. 2015 [1]).

**Table 2:** Diagnosis of dementia: unitary measures of discrimination at various MACE cut-offs.

Cut-off	Acc; Inacc	Y (= Sens + Spec − 1)	PSI (= PPV + NPV − 1)	II (= 2(Acc − 1))	DOR (= LR+/LR−)	SUI (= CUI+ + CUI−)	F Measure (F1 Score)
≤29/30	0.17; 0.83	0.02	0.15	−0.66	∞	0.15	0.27
≤28/30	0.20; 0.80	0.05	0.13	−0.61	6.72	0.21	0.27
≤27/30	0.26; 0.74	0.12	0.16	−0.48	17.3	0.30	0.29
≤26/30	0.37; 0.63	0.21	0.17	−0.33	31.9	0.40	0.31
≤25/30 *	0.42; 0.58	0.31	0.19	−0.17	52.0	0.52	0.34
≤24/30	0.49; 0.51	0.39	0.22	−0.01	38.7	0.64	0.37
≤23/30	0.56; 0.44	0.47	0.24	0.12	52.8	0.74	0.40
≤22/30	0.63; 0.37	0.53	0.27	0.25	47.7	0.82	0.44
≤21/30 *	0.69; 0.31	0.59	0.31	0.37	32.2	0.93	0.48
≤20/30	0.74; 0.26	0.619	0.34	0.48	25.1	1.03	0.51
≤19/30	0.77; 0.23	0.614	0.35	0.54	18.9	1.07	0.53
≤18/30	0.80; 0.20	0.60	0.38	0.60	16.0	1.11	0.55
≤17/30	0.81; 0.19	0.56	0.37	0.62	12.8	1.09	0.54
≤16/30	0.84; 0.16	0.58	0.43	0.68	16.1	1.17	0.58
≤15/30	0.859; 0.14	0.56	0.46	0.72	16.5	1.20	0.59
≤14/30	0.867; 0.13	0.51	0.49	0.74	15.8	1.19	0.57
≤13/30	0.866; 0.13	0.41	0.49	0.73	13.3	1.12	0.52

Acc: correct classification accuracy; DOR: diagnostic odds ratio; II: identification index; Inacc: inaccuracy; PSI: predictive summary index; Sens: sensitivity; Spec: specificity; SUI: summary utility index; Y: Youden index. * Test cut-off established in index study (Hsieh et al. 2015 [1]).

**Table 3:** Diagnosis of dementia: “number needed to” measures (rounded to next highest integer value) and “likelihood to be diagnosed or misdiagnosed” at various MACE cut-offs.

Cut-off	NND (= 1/Y)	NNP (= 1/PSI)	NNM (= 1/Inacc)	NNS (= 1/II)	NNSU (= 1/SUI)	LDM (= NNM/NND; NNM/NNP)
≤29/30	50	7	2	−2	7	0.04; 0.29
≤28/30	20	8	2	−2	5	0.10; 0.25
≤27/30	9	7	2	−3	4	0.22; 0.29
≤26/30	5	6	2	−4	3	0.40; 0.33
≤25/30 *	4	6	2	−7	2	0.50; 0.33
≤24/30	3	5	2	−108	2	0.67; 0.40
≤23/30	3	5	3	9	2	1.00; 0.60
≤22/30	2	4	3	4	2	1.50; 0.75
≤21/30 *	2	4	4	3	2	2.00; 1.00
≤20/30	2	3	4	3	1	2.00; 1.33
≤19/30	2	3	5	2	1	2.50; 1.67
≤18/30	2	3	5	2	1	2.50; 1.67
≤17/30	2	3	6	2	1	3.00; 2.00
≤16/30	2	3	7	2	1	3.50; 2.33
≤15/30	2	3	8	2	1	4.00; 2.67
≤14/30	2	3	8	2	1	4.00; 2.67
≤13/30	3	3	8	2	1	2.67; 2.67

LDM: likelihood to diagnose or misdiagnose; NND: number needed to diagnose; NNM: number needed to misdiagnose; NNP: number needed to predict; NNS: number needed to screen; NNSU: number needed for screening utility. * Test cut-off established in index study (Hsieh et al. 2015 [1]).

**Table 4:** Diagnosis of MCI: paired measures of discrimination at various MACE cut-offs.

Cut-off	Sensitivity (= Recall); Specificity	False Positive (FPR); False Negative (FNR)	PPV (= Precision); NPV	LR+; LR−	CUI+; CUI−
≤29/30	1.00; 0.03	0.97; 0	0.35; 1.00	1.03; 0	0.35; 0.03
≤28/30	1.00; 0.09	0.91, 0	0.37; 1.00	1.09; 0	0.37; 0.09
≤27/30	0.99; 0.20	0.80; 0.01	0.40; 0.99	1.25; 0.02	0.40; 0.20
≤26/30	0.98; 0.32	0.68; 0.02	0.43; 0.96	1.45; 0.07	0.42; 0.31
≤25/30 *	0.95; 0.46	0.54; 0.05	0.48; 0.95	1.76; 0.10	0.46; 0.44
≤24/30	0.90; 0.57	0.43; 0.10	0.53; 0.92	2.11; 0.17	0.48; 0.52
≤23/30	0.82; 0.64	0.36; 0.18	0.55; 0.87	2.29; 0.29	0.45; 0.56
≤22/30	0.73; 0.72	0.28; 0.27	0.58; 0.84	2.63; 0.37	0.42; 0.60
≤21/30 *	0.64; 0.79	0.21; 0.36	0.61; 0.80	2.99; 0.46	0.39; 0.63
≤20/30	0.54; 0.84	0.16; 0.46	0.63; 0.77	3.33; 0.55	0.34; 0.65
≤19/30	0.47; 0.88	0.12; 0.53	0.67; 0.76	3.81; 0.60	0.31; 0.67
≤18/30	0.37; 0.89	0.11; 0.63	0.65; 0.73	3.56; 0.70	0.24; 0.65

LR+; LR− = positive and negative likelihood ratio; CUI+; CUI− = positive and negative clinical utility index. * = test cut-off established in index study (Hsieh et al. 2015 [1]).

**Table 5:** Diagnosis of MCI: unitary measures of discrimination at various MACE cut-offs.

Cut-off	Acc; Inacc	Y (= Sens + Spec − 1)	PSI (= PPV + NPV − 1)	II (= 2(Acc − 1))	DOR (= LR+/LR−)	SUI (= CUI+ + CUI−)	F Measure (F1 Score)
≤29/30	0.37; 0.63	0.03	0.35	−0.26	∞	0.38	0.52
≤28/30	0.40; 0.60	0.09	0.37	−0.20	∞	0.46	0.54
≤27/30	0.48; 0.52	0.19	0.39	−0.05	55.4	0.60	0.57
≤26/30	0.55; 0.45	0.30	0.39	0.10	20.9	0.73	0.60
≤25/30 *	0.63; 0.37	0.41	0.43	0.26	17.9	0.90	0.64
≤24/30	0.69; 0.31	0.47	0.45	0.37	12.2	1.00	0.67
≤23/30	0.70; 0.30	0.46	0.42	0.41	8.00	1.01	0.66
≤22/30	0.73; 0.27	0.45	0.42	0.45	7.13	1.02	0.65
≤21/30*	0.73; 0.27	0.43	0.41	0.47	6.45	1.02	0.62
≤20/30	0.73; 0.27	0.38	0.40	0.47	6.07	0.99	0.58
≤19/30	0.74; 0.26	0.35	0.43	0.47	6.33	0.98	0.55
≤18/30	0.71; 0.29	0.26	0.38	0.43	5.09	0.89	0.48

Acc: correct classification accuracy; DOR: diagnostic odds ratio; II: identification index; Inacc: inaccuracy; PSI: predictive summary index; SUI: summary utility index; Y: Youden index. * Test cut-off established in index study (Hsieh et al. 2015 [1]).

**Table 6:** Diagnosis of MCI: “number needed to” measures (rounded to next highest integer value) and “likelihood to be diagnosed or misdiagnosed” at various MACE cut-offs.

Cut-off	NND (= 1/Y)	NNP (= 1/PSI)	NNM (= 1/Inaccuracy)	NNS (= 1/II)	NNSU (= 1/SUI)	LDM (= NNM/NND; NNM/NNP)
≤29/30	34	3	2	−4	3	0.06; 0.67
≤28/30	12	3	2	−6	3	0.17; 0.67
≤27/30	6	3	2	−21	2	0.33; 0.67
≤26/30	4	3	3	10	2	0.75; 1.00
≤25/30 *	3	3	3	4	2	1.00; 1.00
≤24/30	3	3	4	3	1	1.33; 1.33
≤23/30	3	3	4	3	1	1.33; 1.33
≤22/30	3	3	4	3	1	1.33; 1.33
≤21/30 *	3	3	4	3	1	1.33; 1.33
≤20/30	3	3	4	3	2	1.33; 1.33
≤19/30	3	3	4	3	2	1.33; 1.33
≤18/30	4	3	4	3	2	1.00; 1.33

LDM: likelihood to diagnose or misdiagnose; NND: number needed to diagnose; NNM: number needed to misdiagnose; NNP: number needed to predict; NNS: number needed to screen; NNSU: number needed for screening utility. * Test cut-off established in index study (Hsieh et al. 2015 [1]).

**Table 7:** MACE predictive values at differing disease prevalence of dementia and MCI (0.05–0.4).

	Prevalence					
	0.05	0.1	0.15 (Observed)	0.2	0.29 (Observed)	0.4
PPV dementia (cut-off ≤20/30)	0.14	0.26	0.36	0.44	-	0.68
NPV dementia (cut-off ≤20/30)	0.99	0.99	0.98	0.97	-	0.92
PPV MCI (cut-off ≤24/30)	0.10	0.19	-	0.34	0.53	0.58
NPV MCI (cut-off ≤24/30)	0.99	0.98	-	0.96	0.92	0.90

## References

[1] Hsieh S., McGrory S., Leslie F., Dawson K., Ahmed S., Butler C.R., Rowe J.B., Mioshi E., Hodges J.R. (2015). The Mini-Addenbrooke’s Cognitive Examination: A new assessment tool for dementia. Dement. Geriatr. Cogn. Disord..

[2] Matias-Guiu J.A., Fernandez-Bobadilla R. (2016). Validation of the Spanish-language version of Mini-Addenbrooke’s Cognitive Examination as a dementia screening tool [in Spanish]. Neurologia.

[3] Larner A.J. (2015). Mini-Addenbrooke’s Cognitive Examination: A pragmatic diagnostic accuracy study. Int. J. Geriatr. Psychiatry.

[4] Williamson J.C., Larner A.J. (2018). MACE for diagnosis of dementia and MCI: 3-year pragmatic diagnostic test accuracy study. Dement. Geriatr. Cogn. Disord..

[5] Larner A.J. (2019). Free-Cog: Pragmatic test accuracy study. Dement. Geriatr. Cogn. Disord..

[6] Doane D.P., Seward L.E. (2011). Measuring skewness: A forgotten statistic?. J. Stat. Educ..

[7] Youden W.J. (1950). Index for rating diagnostic tests. Cancer.

[8] Linn S., Grunau P.D. (2006). New patient-oriented summary measure of net total gain in certainty for dichotomous diagnostic tests. Epidemiol. Perspect. Innov..

[9] Mitchell A.J., Kattan M.W. (2009). Index test. Encyclopedia of Medical Decision Making.

[10] Larner A.J. (2015). Optimizing the cutoffs of cognitive screening instruments in pragmatic diagnostic accuracy studies: Maximising accuracy or Youden index?. Dement. Geriatr. Cogn. Disord..

[11] O’Caoimh R., Gao Y., Svendovski A., Gallagher P., Eustace J., Molloy D.W. (2017). Comparing approaches to optimize cut-off scores for short cognitive screening instruments in mild cognitive impairment and dementia. J. Alzheimers Dis..

[12] Habibzadeh F., Yadollahie M. (2013). Number needed to misdiagnose: A measure of diagnostic test effectiveness. Epidemiology.

[13] Larner A.J. (2018). Number needed to diagnose, predict, or misdiagnose: Useful metrics for non-canonical signs of cognitive status?. Dement. Geriatr. Cogn. Dis. Extra.

[14] Larner A.J. (2019). Evaluating cognitive screening instruments with the “likelihood to be diagnosed or misdiagnosed” measure. Int. J. Clin. Pract..

[15] Larner A.J. (2019). Manual of Screeners for Dementia: Pragmatic Test Accuracy Studies.

[16] Metz C.E. (1978). Basic principles of ROC analysis. Semin. Nucl. Med..

[17] Walter S.D. (2002). Properties of the summary receiver operating characteristic (SROC) curve for diagnostic test data. Stat. Med..

[18] Davis J., Goadrich M. (2006). The relationship between Precision-Recall and ROC curves. ICML ’06: Proceedings of the 23rd International Conference on Machine Learning.

[19] Saito T., Rehmsmeier M. (2015). The precision-recall plot is more informative than the ROC plot when evaluating binary classifiers on imbalanced datasets. PLoS ONE.

[20] Powers D.M.W. (2011). Evaluation: From precision, recall and F-measure to ROC, informedness, markedness and correlation. J. Mach. Learn. Technol..

[21] Cohen J. (1988). Statistical Power Analysis for the Behavioral Sciences.

[22] Sawilowsky S.S. (2009). New effect sizes rules of thumb. J. Mod. Appl. Stat. Methods.

[23] Davis D.H., Creavin S.T., Noel-Storr A., Quinn T.J., Smailagic N., Hyde C., Brayne C., McShane R., Cullum S. (2013). Neuropsychological tests for the diagnosis of Alzheimer’s disease dementia and other dementias: A generic protocol for cross-sectional and delayed-verification studies. Cochrane Database Syst. Rev..

[24] Larner A.J. (2015). The Q* index: A useful global measure of dementia screening test accuracy?. Dement. Geriatr. Cogn. Dis. Extra.

[25] Nasreddine Z.S., Phillips N.A., Bédirian V., Charbonneau S., Whitehead V., Collin I., Cummings J.E., Chertkow H. (2005). The Montreal Cognitive Assessment, MoCA: A brief screening tool for mild cognitive impairment. J. Am. Geriatr. Soc..

[26] Larner A.J. (2017). MACE versus MoCA: equivalence or superiority? Pragmatic diagnostic test accuracy study. Int. Psychogeriatr..

[27] Ozenne B., Subtil F., Maucort-Boulch D. (2015). The precision-recall curve overcame the optimism of the receiver operating characteristic curve in rare diseases. J. Clin. Epidemiol..

[28] Keilwagen J., Grosse I., Grau J. (2014). Area under precision-recall curves for weighted and unweighted data. PLoS ONE.

